# Structured Cluster Detection from Local Feature Learning for Text Region Extraction

**DOI:** 10.3390/e25040658

**Published:** 2023-04-14

**Authors:** Huei-Yung Lin, Chin-Yu Hsu

**Affiliations:** 1 Department of Computer Science and Information Engineering, National Taipei University of Technology, Taipei 106, Taiwan; 2 Department of Electrical Engineering, National Chung Cheng University, Chiayi 621, Taiwan

**Keywords:** machine vision, structure pattern analysis, text region detection

## Abstract

The detection of regions of interest is commonly considered as an early stage of information extraction from images. It is used to provide the contents meaningful to human perception for machine vision applications. In this work, a new technique for structured region detection based on the distillation of local image features with clustering analysis is proposed. Different from the existing methods, our approach takes the application-specific reference images for feature learning and extraction. It is able to identify text clusters under the sparsity of feature points derived from the characters. For the localization of structured regions, the cluster with high feature density is calculated and serves as a candidate for region expansion. An iterative adjustment is then performed to enlarge the ROI for complete text coverage. The experiments carried out for text region detection of invoice and banknote demonstrate the effectiveness of the proposed technique.

## 1. Introduction

Due to the prevalence and availability of imaging devices, the use of computer vision techniques is becoming popular in our daily lives. Many objects of image recognition, such as human face, fingerprint and traffic sign, have been extensively investigated as research topics over the past few decades. The detection of regions of interest (ROIs) in images is thus a very important preprocessing stage [1,2]. It is commonly adopted to identify the image region that is meaningful to human perception. Since further analysis can then be carried out for scene-understanding tasks, ROI detection is usually considered as an early stage for extraction of information from acquired images [3]. In general, the overall performance of a machine perception system is highly reliant on the correctness of the ROI detection results.

From the perspective of visual perception, ROI is a fairly general term, and the definition is rather diverse depending on the application scenario [4]. It could represent a variety of pattern classes ranging from natural beings to man-made structures. As an example, the characteristics of images features utilized for the detection of human face and traffic symbols are very different [5,6]. Thus, the methodologies for the extraction of ROIs usually take the identification of some specific pattern structures into consideration. The features for pattern analysis might be manually extracted, using low-level image properties, or derived from learning-based techniques. However, depending on the amount of available training data, encoding high-level features via learning is generally not a simple task. In addition, more computational resources will be required for model training and testing.

To identify the regions of interest based on low-level image properties, local feature analysis is commonly used to obtain the correspondences between the reference and target images [7]. The histogram of oriented gradients (HOG) is a common feature to compute the gradient distributions of the objects. The features can then be adopted by a linear support vector machine (SVM) classifier for pedestrian detection [8]. To deal with the different scales of the targets, Marques et al. adopted size-invariant local features for marine vessel detection [9]. Based on saliency analysis, Achanta et al. presented a frequency-tuned approach to compute salient regions in images using low-level features of color and luminance [10]. It was capable of deriving full-resolution saliency maps with meaningful boundaries. However, the proposed method was mainly utilized to analyze natural scene images. Some good detection results can be obtained using the above approaches, and they have implicitly assumed that the target region is continuous and smooth in the image. The algorithms have not been directly adopted for general cases, where regions contain isolated internal structures.

In this work, we are interested in the detection of structured regions with isolated internal patterns. More specifically, this could be a text region with arbitrary orientation in different scenarios. Due to the wide availability of text descriptions in our living environment, text detection is usually the first step toward scene understanding. In early works, Epshtein et al. [11] converted edge gradients to the width of handwriting texts and used the distribution to localize the text region. A visual attention model was adopted to investigated the feasibility to video applications for salient object detection [12]. The strong signals associated with texts can then be accordingly extracted. To detect the texts in natural scenes, Yin et al. proposed a technique to extract maximally stable extremal regions as the character candidates for grouping [13]. The text classification is performed based on the posterior probability of the text candidates estimated by a character classifier. Most current developments consider the text region as an integrated part for detection, and the algorithms focus on extracting the regional features while minimizing the text localization error for identification.

**Figure 1:**
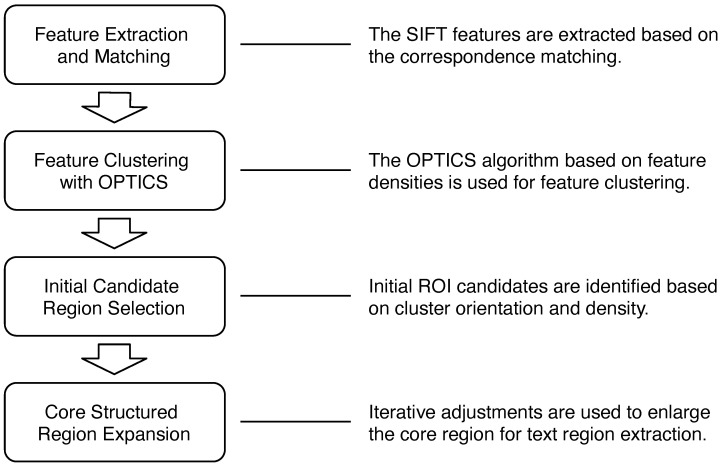
The four stages of our proposed structured region detection technique. It consists of feature extraction and matching, feature clustering, initial region selection and core structured region extraction.

This paper presents a structured region detection approach based on the distillation of local image features with clustering analysis. We are focused on the extraction of structured clusters from local feature learning. Thus, the objective is not for a general character recognition task. Figure 1 depicts the system flow of the proposed technique. The features in the target image corresponding to the similar structures in the reference images are first extracted, followed by region detection from analysis of the clustering characteristics of the ordered feature points. In our proposed method, the images with multiple characters in the database are used for feature matching. It is able to detect the text clusters under the sparsity of feature points derived from some characters. The location with high feature density is selected as a candidate, and an iterative process is carried out to increase the ROIs for the derivation of some suitable region with structured content. Since fast detection using limited computational resources is the key to the success of real-world applications, it is desirable to reduce the costs of model training and online inference. Different from existing deep neural network approaches, our technique can be easily implemented with hardware-oriented acceleration [14,15]. In the experiments, the text detection and recognition of invoice and banknote have demonstrated the effectiveness of the proposed technique.

The main contributions of this paper are as follows.

- A new approach based on correspondence extraction and clustering analysis of local features is proposed for structured region detection.
- A multi-stage algorithm with robust receptor descriptor is presented for character recognition.
- The proposed technique is capable of fast region detection with limited computational resources and can be easily implemented with hardware acceleration.

## 2. Related Works

The investigation of text detection and recognition has been conducted over the decades. It is important due to the necessity of visual communication in the human-centered environment. The techniques are commonly developed based on the application scenarios and can be divided into text region extraction for documents (such as banknotes and invoices) and general scenes. For outdoor environments, the regions of interest usually cover a variety of scene texts, which includes signboards, license plates and digital traffic boards, etc. There also exist some other issues such as language and orientation. These might require the detection of more general structured patterns instead of performing template matching using prior knowledge. Recently, subspace clustering has been developed for various image analysis tasks, including sparse clustering applied to hyperspectral images [16]. It is also used to deal with multi-view data clustering [17] and multivariate time series data [18], and promising results have been reported.

The proper extraction of texts and numbers is the key to automatic document processing and analysis. Different from text detection in general scenes or handwritten documents [19], the pattern structures are usually more constrained in terms of size and format. For banknote recognition applications, Dittimi et al. presented a technique based on multi-class SVM [20]. The classification is carried out via the principal component analysis of HOG features. In [21], Pham et al. proposed a method based on discriminative region selection using the masks derived from a similarity map. The genetic algorithm was then applied out to optimize the banknote regions. More recently, a machine learning-based approach for simultaneous ROI extraction and character classification was presented [22]. Based on the use of knowledge distillation, the complexity can be reduced with a simple model for fast computation.

The technical process for identification of invoice information shares similarities with that of banknote recognition in pattern structure detection. However, the extraction of invoice numbers is usually more complicated due to the variation of background texture. To identify invoice information, Sun et al. proposed a template-based method for region detection [23]. Optical character recognition is then carried out to retrieve the text information. Tian et al. developed an iterative self-learning framework for intelligent financial ticket recognition [24]. A network model was constructed based on Faster R-CNN to recognize multiple ticket formats. In their work [25], Jiang et al. proposed a unified framework to process and recognize VAT invoices. The end-to-end model was trained to handle challenging cases with multi-oriented texts.

Among the text detection and recognition application scenarios, extracting the information for general outdoor scenes is the most challenging task. They contain a wide variety of text arrangements, sizes, styles, etc. In their early works, Coates et al. developed a text detection and recognition system based on a scalable learning algorithm [26]. A band of image features is learned from unlabeled data, followed by a linear classifier used for scene text extraction. Wang et al. [27] presented a texture-based approach for text detection, where a scale-insensitive adaptive region proposal network is first used to create text proposals, followed by a local orthogonal texture-aware method to represent the text. Zhang et al. [28] emphasized application in urban scenes and proposed a deep neural network approach for intelligent transportation systems. A keyword search tool was combined with a GIS system for street scene textual indexing. In [29], Yao et al. presented a unified framework for detecting multi-oriented scene text. A dictionary search-based method was proposed to correct character recognition errors. To deal with multi-lingual scene texts, the ICDAR reading challenge was conducted on the image datasets containing 10 languages [30]. The text structure feature extractor was used to simulate the Chinese text human cognition model [31]. In the recent adversarial learning method, Zhan et al. proposed a geometry aware domain adaption network [32]. It is able to synthesize multiple adapted images with different viewpoints for scene text detection.

In the existing literature, there are not many works focused on the detection of general structured patterns. Compared to the semantic information used for specific applications, low-level features are better suited for structured region detection. Based on the idea of saliency detection, Li et al. proposed a method to measure the ‘characterness’ of a region [33]. It was constructed using a Bayesian framework to integrate the text region by exploiting the dependencies among the characters. Zhu et al. presented a low-level detector based on MSER and region proposal for text detection [34]. The heuristic features are then used to group the characters into text lines. To ensure that structured pattern detection can be adopted to different high-level image-understanding tasks, it should be able to provide local clustering with a globally consistent scale.

## 3. Feature Selection and OPTICS Clustering

**Figure 2:**
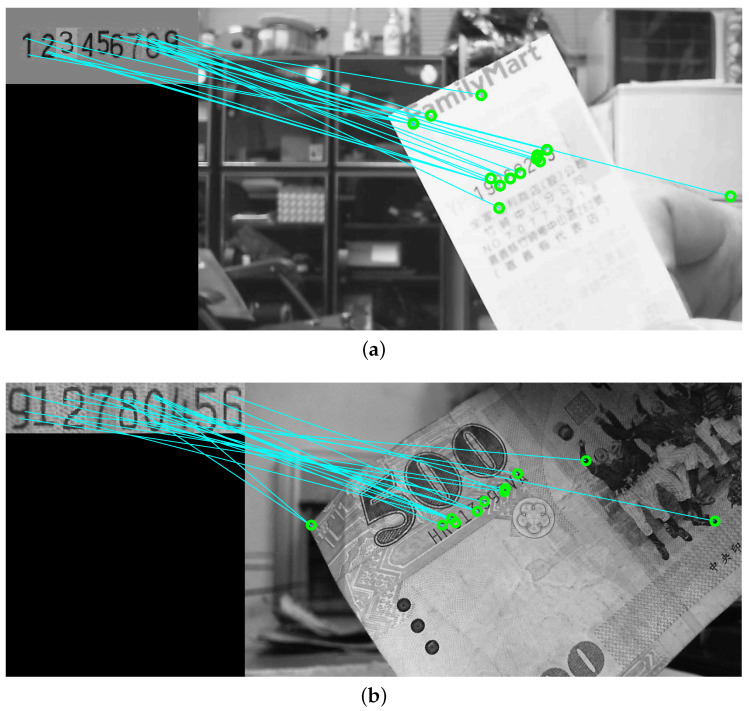
The results of feature correspondence matching between the target image and a database template. Our objective is to find the candidate feature locations based on the pre-established structures of interest. (**a**) An invoice correspondence matching result. (**b**) A banknote correspondence matching result.

In the proposed structured region detection pipeline, the first step is to extract the feature points in the target image. These points should possess properties similar to those of the reference images in the database. As in the examples illustrated in Figure 2, the objective is to find the candidate feature locations based on pre-established structures of interest. To perform correspondence matching, the commonly used SIFT descriptor is adopted in this work for feature extraction. In most applications, it is used for object detection or scene matching from different viewpoints. The correspondences between the reference and target image features are established to derive the homography transformation. For our use of structured pattern extraction, the text regions for detection in the images are relatively small. The number of feature points for correspondence matching is very limited. Thus, it is not feasible to use to the distribution of feature points for region extraction, because a large amount of data is generally needed to increase the features for pattern identification.

One important property of the structured region of interest is the spatial proximity of individual building elements. Thus, our idea is to aggregate the few matching correspondences of each element to form the rough region clusters for detection. To maintain the stability of region detection via the aggregation of feature points, it is expected that as many feature correspondences as possible are extracted for each element. However, the increasing number of images for feature matching implies a higher computational cost, which is usually not preferable for the development o real-time systems. Therefore, in addition to the mismatching rate and the storage for reference images, one also needs to consider the computation time when performing the feature matching task.

To achieve robust feature matching between the target image and reference data, internal feature correspondence extraction among the training images is first conducted. This serves as a training stage for the application-specific feature selection. A correspondence matching is carried out for the SIFT features in all reference images, and the points with good pairing are considered as important features. To be more specific, suppose there are *N* images in the training dataset, and $p_{i}$ is a feature point which belongs to the ROI of the *i*th image. Let

(1) $$S_{i}=\left\{\left(p_{i},p_{j}\right)\;|\;1\leq j\leq N,j\neq i\right\}$$

for 1≤i≤N, where $\left(p_{i},p_{j}\right)$ denotes the correspondence between image *i* and *j* if it exists. Then the point $p_{i}$ is defined as a *prominent feature* if the number of correspondence pairs is greater than a preset threshold, or $|S_{i}|\geq T$.

**Figure 3:**
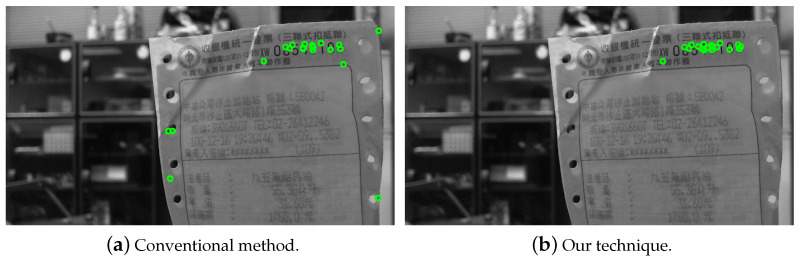
The feature extraction results using (**a**) the conventional method and (**b**) our technique. The algorithm can reject undesired points while keeping the important features for region detection. It can be seen that our method is able to reject the undesired points while allowing the important features for region detection to remain intact.

It should be noted that, depending on the feature extraction criteria, there could be zero to many prominent feature points for a reference image in the training data. The images without any prominent features can then be removed from the dataset. Only the prominent features in the reference images are used to match the features in the target images for region detection. To improve the matching efficiency, one-to-many feature correspondences between the target and reference images are also allowed. This strategy to increase the number of feature points is not applicable to most applications utilizing feature correspondence matching. The feasibility of one-to-many mapping is built upon the use of the certified features derived from the database images. Figure 3a,b show the feature extraction results using the conventional method and the proposed technique, respectively. It can be seen that our method is able to reject the undesired points while allowing the important features for region detection to remain intact.

After feature extraction and matching, the next stage is to identify the feature points scattered within the structured image region. It is necessary to analyze the spatial relationship among these feature points, and generate dense clusters as candidate regions for ROI detection. However, due to the possible outliers in the set of image features, it is mandatory to perform effective clustering to localize the core feature points to form an initial detection region. The development of a robust clustering method is very important, since the results of candidate detection will be significantly affected by outlier features.

**Figure 4:**
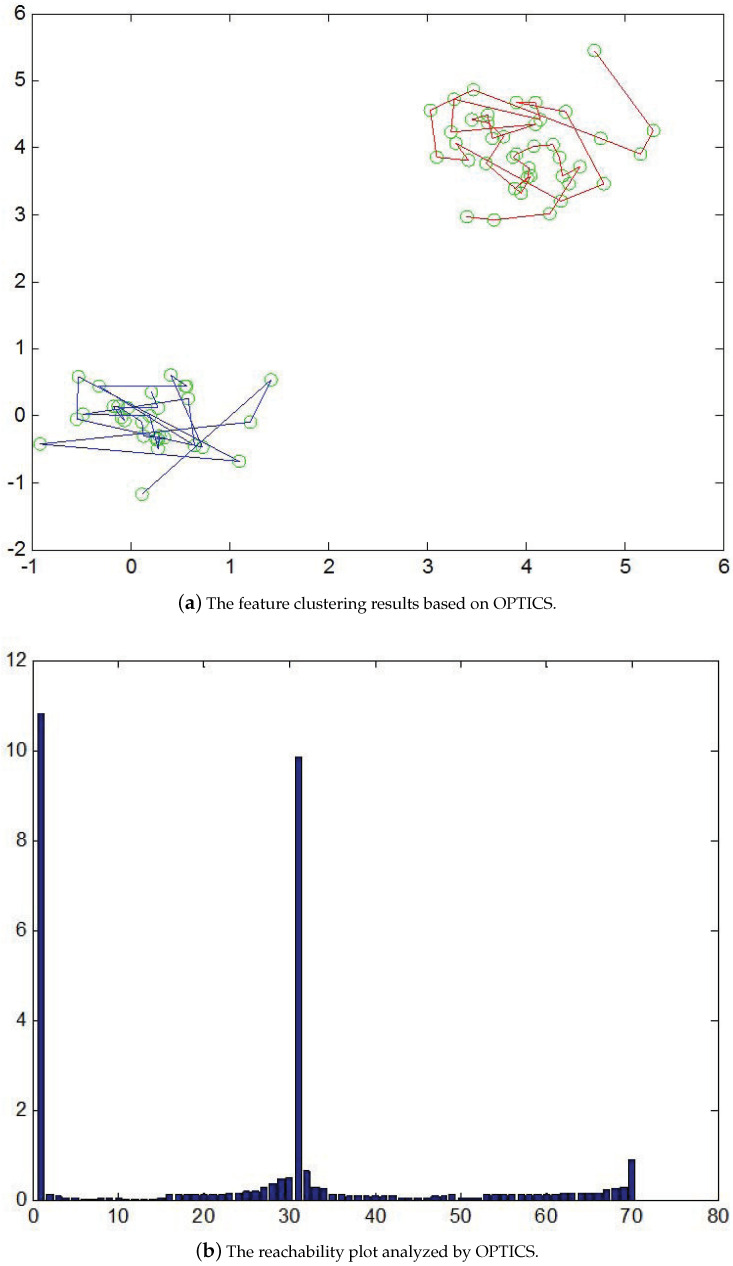
An illustration of the hierarchical feature clustering technique based on the OPTICS algorithm. (**a**) Two sets of dense feature points are located on the corners. (**b**) The two flat regions in the intervals (1,30) and (31,70) associated with two clusters are indicated.

In general, it is required that good parameter settings be provided for clustering analysis algorithms [35]. However, it is not generally the case that there is an internal data structure that can be described clearly using a set of global parameters. The proper parameters are not only difficult to derive but also sensitive to the clustering results. In this work, we present a hierarchical clustering technique for feature aggregation based on the OPTICS (ordering points to identify the clustering structure) algorithm [36]. It does not directly perform the clustering but provides a feature sorting scheme to represent the data. Based on the ordering of reachability distances associated with the feature density, a reachability plot is generated and used for cluster identification. Through the analysis of cluster densities, it is possible to maintain stable feature structures using a wide range of parameters. Figure 4 illustrates a typical example of OPTICS clustering. Two sets of dense feature points located on the corners are shown in Figure 4a. The reachability plot as depicted in Figure 4b indicates the two flat regions in the intervals (1,30) and (31,70) are associated with two clusters in Figure 4a. In other words, the peak at around 31 indicates the density-based distance is large and can be adopted for the cluster derivation.

**Figure 5:**
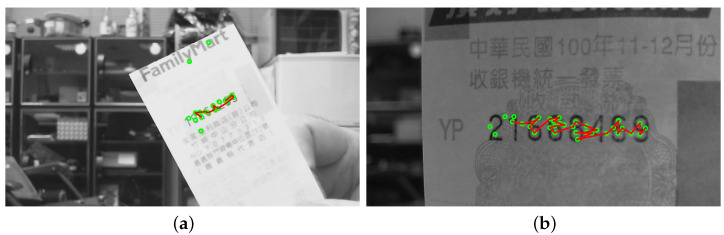
The OPTICS feature clustering results of the input images with two different scales. The core detection regions are indicated by the connected segments in red for (**a**) small-scale image input; (**b**) large-scale image input.

When clustering is performed using a large amount of data, the loss of clusters due to improper parameter settings is less likely. In case there are only a small number of features in the target images for correspondence searching, the final results will be more sensitive to the clustering approaches. Therefore, the OPTICS algorithm is further modified to accommodate extra constraints on the feature distribution to make it more robust under the image scale change. If the density of feature points is less than a preset threshold, the image will be normalized for further hierarchical clustering. The clustering results with two different scales are illustrated in Figure 5, where the core detection regions are indicated by the connected segments in red.

## 4. Structured Region Extraction

Given the results of feature extraction and clustering, a rough candidate region can be obtained. The next stage is to identify the precise ROI for a specific application, including translation, rotation and scaling, according to its pattern structure. To detect a structured region with certain characteristics, it is necessary to use the known templates for learning and pattern analysis. The bounding box with the best structural fitting is then derived by adjusting the candidate region from matching and validation iteratively. Thus, the application-specific ROI templates are extracted from the training images, followed by analyzing the structural characteristics for region identification and validation on the testing data.

**Figure 6:**
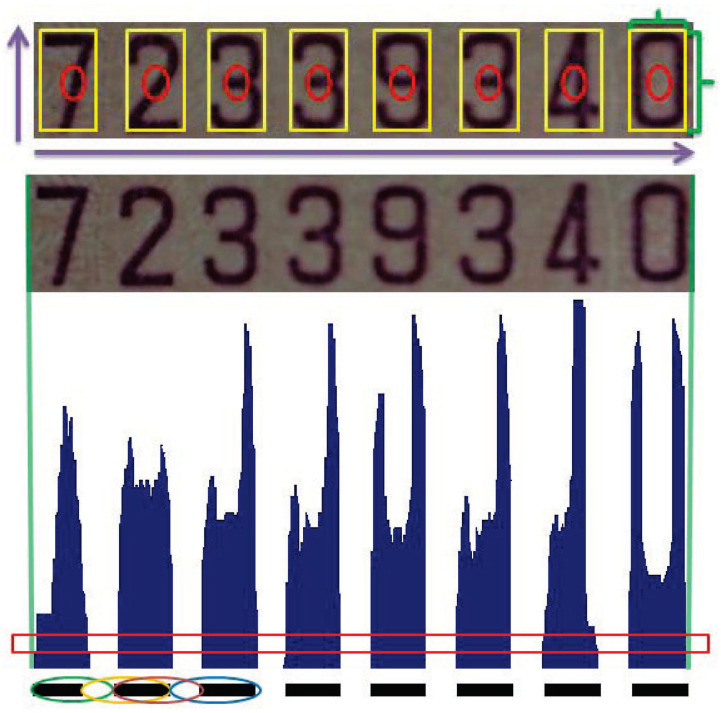
A typical case of an invoice containing eight digits. The structural properties, including the alternation of character and letter-spacing, are adopted for pattern identification. In addition to the structural properties, the alternation of character and letter-spacing is also adopted for pattern identification.

In scene text detection, the ROI to be identified usually possesses a similar type of structure in the image. Therefore, it is possible to perform the region extraction using the strong relations among the elements. As an example, a serial number of an invoice or a banknote is composed of several digits and characters. The fixed structural properties include the number of elements in the ROI, the space between the elements, and the aspect ratio of ROI and individual elements. This provides the important information for the extraction of proper regions of interest. Figure 6 illustrates a typical example of an invoice containing 8 digits. In addition to the structural properties, the alternation of character and letter-spacing is also adopted for pattern identification. Since the width ratio between character and letter-spacing is both scale- and space-invariant, it can be used as a stable feature for matching.

In the implementation, the vertical projection of the region of interest is used to derive the histogram of character pixels as shown in Figure 6. Let the widths of character and letter-spacing be denoted by $c_{2i-1}$ and $s_{2j}$, for i=1,2,⋯,N and j=1,2,⋯,N−1, respectively. *N* is the total number of characters. To increase the robustness of pattern matching, a series of width ratios derived from the neighboring character and letter-spacing is used. That is, a feature vector coded by

$${\left(\frac{c_{1}}{s_{2}},\frac{c_{3}}{s_{4}},\cdots ,\frac{c_{2N-3}}{s_{2N-2}},\frac{c_{2N-1}}{s_{2N-2}}\right)}^{⊤}$$

is used for the template matching. Since the degree of letter-spacing is one less than character, the denominator of the last entry is repeated.

**Figure 7:**
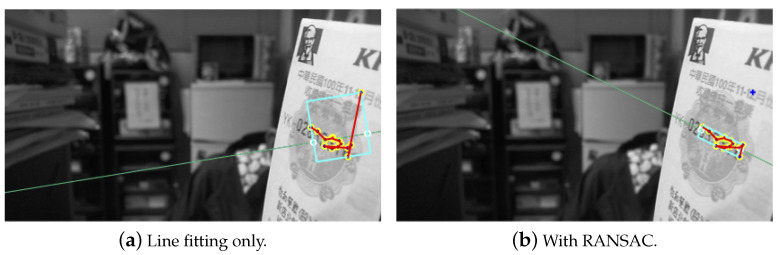
The core region detection based on the clustering results with (**a**) only line fitting and (**b**) additional RANSAC for outlier removal.

The feature selection and clustering analysis have allowed the identification of a cluster in a specific region. This is required to extract the candidate region as close to the true location as possible based on the clustering result. This will facilitate the adjustment of region estimation in the next stage. In serial number extraction, a series of elements are arranged along a straight line, and the feature point should be found in the fixed orientation. Consequently, applying a line-fitting algorithm carried to the feature cluster will allow identifying the features scattered along the text direction. An initial ROI derived from the rectangular region containing the image features can then be constructed. This will serve as the core region for enlargement along the text direction to include all characters in a later stage. An example of core region detection is illustrated in Figure 7a, where the bounding box might contain some outliers and the orientation is not correctly obtained.

**Figure 8:**
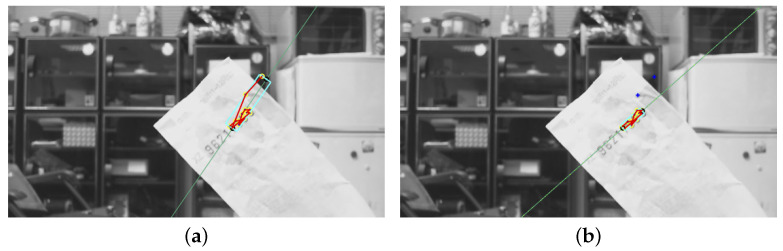
Due to the way in which the ordered feature strings are constructed, the outliers commonly appear at the two endpoints. The outlier rejection for the feature points along the text direction based on the distance distribution obtained from OPTICS clustering: (**a**) OPTICS clustering; (**b**) with outlier rejection.

To make the core region extraction more robust, two constraints are adopted to remove the outliers of a cluster. First, the feature points further away from the initial ROI obtained based on the line model are eliminated using RANSAC. Figure 7b depicts the outlier removal results for Figure 7a. Nevertheless, the outlier features along the text direction will still be preserved under this condition, as in the example shown in Figure 8a. Another criterion for outlier rejection is based on the density correlation of ordered feature points obtained from the OPTICS algorithm. Due to the way in which the ordered feature strings are constructed, the outliers commonly appear at the two endpoints. Furthermore, the distances to the connecting feature points are significantly larger than the rest. Thus, a thresholding process is performed on the distance distribution of feature points to reject the outliers based on the variation. Figure 8b shows the filtering result of the outliers in the text direction of Figure 8a.

The next stage is to adjust the region candidate to correctly match the text region of interest. Our objective is to make the adjustment feasible whenever the candidate location is inside the text region, regardless of the orientation accuracy and the size difference. The procedure will be iteratively carried out, until the extracted region is satisfactory for text detection. First, the image is rotated with respect to the image scanline according to the orientation obtained from the feature point distribution of the region candidate. The purpose of this step is to initialize a rectangular bounding box to enclose the candidate region and use it as the core for expansion. Based on prior knowledge of the specific structures of interest, it is possible to enlarge or shift the bounding box if the projections of connected components in the horizontal or vertical direction are substantially different from the expected region specification. If the border of the current candidate region comes across some characters, this implies that the text is not completely covered by the ROI. Consequently, a region expansion process will be iteratively performed until the characteristics of the text region are satisfied.

To determine the shift direction, we consider the distribution of connected components and the regions formed by the horizontal and vertical projections of the ROI. The direction involves less blank areas and indicates a high possibility of more components to be identified. Since there could be erroneous results due to an inaccurate initial region assessment, timely adjustment is required to avoid the accumulation of improper expansions of the detected region. The rotation for the ROI is determined according to the accumulation error derived from the connected components and refer to an element in the region with an average size. Due to the presence of noise, the height difference between the reference and the remaining elements is used for the assessment of the accumulation error. The ROI will be rotated using the angle derived based on the error if it exceeds the threshold. In addition, the selected reference element is also used for severe noise filtering, since the object sizes are generally similar in the text region. The algorithm for ROI extraction, including the iterative process and region rotation, is depicted in Algorithm 1.
**Algorithm 1** ROI Extraction Algorithm**Require:** The initial region candidate.**Ensure:** The text region of interest.  1:*ROI*← RotateByFirstOrientation(*candidate ROI*)  2:*iterative*← True  3:WHILE *iterative*  4:   *iterative* ← False  5:   Binarization(*ROI*)  6:   (*midAreaCC, quantity*) ←  7:        ConnectedComponentAnalysis(*ROI*)  8:   IF *heightAccError* > *heightRatio*  9:        Rotate(*ROI*)  10:      Truncate(*ROI*)  11:      *iterative* ← True  12:      continue  13:   IF *quantity* < *totalAmount*  14:      IF margin is confused  15:         *trend* ← ComputePointDistributed()  16:         ExpandByTrend(*ROI*) or  17:         ShiftByTrend(*ROI*)  18:      ELSE  19:         ExpandByMargin(*ROI*) or  20:         ShiftByMargin(*ROI*)  21:      *iterative* ← True  22:END WHILE  23:return ROI

## 5. Character Recognition

In this work, we propose a multi-stage approach for character recognition using a neural network. Most of current algorithms require significant training time to iteratively optimize the performance. However, the training time generally grows exponentially in proportion to the amount of training data. One simple method to cope with this problem is to divide the training dataset to a number of smaller subsets. Consequently, the overall training time can be derived by the largest training subset. Based on a similar concept, a preprocessing stage is applied to group characters with the same properties. A fast training network is then developed for the recognition of diverse characters.

The training data are divided into groups in our recognition framework based on the character symmetry properties and the Euler number. Multiple neural networks are used for fast learning and inference. When performing the recognition, no preprocessing is carried out on the input characters. It is not required that a specific network be used according to the associated group. Each character is taken as an input to all networks for processing, and the best three recognition results are selected as the candidates for verification in the second stage.

In the proposed multi-stage character recognition scheme, a similarity evaluation is performed in the second stage using pixelwise comparison. The input characters are compared with the first-stage results from all groups. Let $s_{i}$ denote the similarity metric defined by the pixelwise region intersection with the *i*th group. The final result is then determined by the score $\alpha_{i}s_{i}$, where $\alpha_{i}$ is a weight factor. If the input character possesses the same Euler number with the *i*th group, then set $\alpha_{i}>1$ to provide a high similarity weight. Otherwise, we let $\alpha_{i}=1$. The first-stage output that has the highest weighted score is taken as the final recognition result.

**Figure 9:**
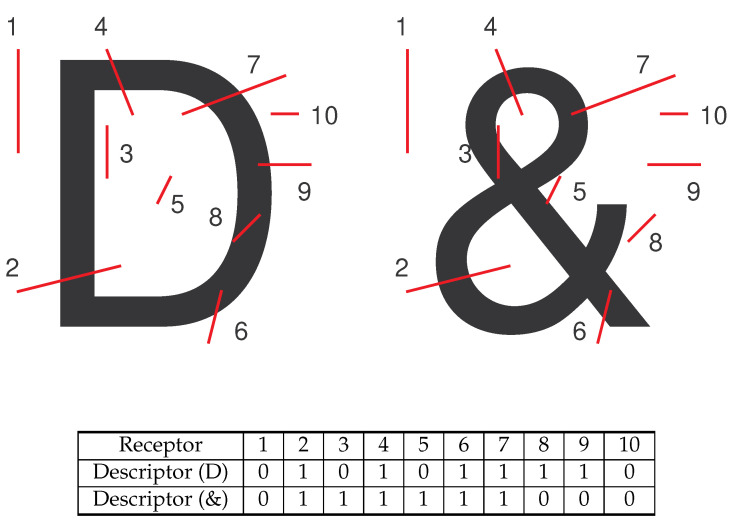
Two examples of the descriptors derived with 10 receptors applied on the characters ‘D’ and ‘&’. The values 1 and 0 in the table indicate the presence of intersection with the character.

The character recognition system using conventional neural networks generally takes the pixel values of the images for processing. It is sensitive to character deformation and image noise due to the spatial relations among the characters in the input layer. In this paper, we present a method to extract more robust descriptors from the character using random *receptors*. This is designed to reduce the influence of image normalization for the neural network. The basic idea is to drop some random line segments generated with various orientations and lengths on the images and record the status of intersection between the character and different receptors. Figure 9 illustrates two examples of the descriptors derived with 10 receptors applied on the characters. The values 1 and 0 in the table indicate the presence of intersection with the character.

Although the receptors can be manually constructed using a set of parameters, they are usually randomly generated with only the number specified. Since it is not possible to guarantee the uniqueness of feature descriptors derived from characters, they are treated as as additional nodes in the input layer of the network for recognition. This network structure design is able to improve the stability of character recognition results under the influence of image deformation between the training and testing data. In our current implementation, the receptors are adopted for the first-stage recognition network. The same idea might be applied to the second-stage network to transform the character recognition problem to the similarity evaluation of binary codes. This approach can be further investigated, albeit its advantage to the recognition system is not clear.

## 6. Experiments and Evaluation

In the experiments, the proposed method is carried out on real-world images for structured region detection. Three application scenarios, including text detection for invoices and serial number identification for banknotes, are adopted for performance evaluation. Investigated in the tests are feature matching, feature clustering, region selection, and character recognition. Since all of these stages are highly correlated, we also tabulate the intermediate results for analysis. Compared to the region extraction algorithms based on deep neural networks, the implementation of our technique is simple and easy to use. It does not require a large image dataset for training or high computational power. To ensure the approach is suitable for practical applications, the testing samples are collected in a cluttered environment. The number of images and the contained regions of interest for the different applications are shown in the first and second rows of Table 1. Since the number of regions for detection is not constrained, there might exist multiple regions of interest, as indicated in the table. The region detection results are then used for performance evaluation.

**Figure 10:**
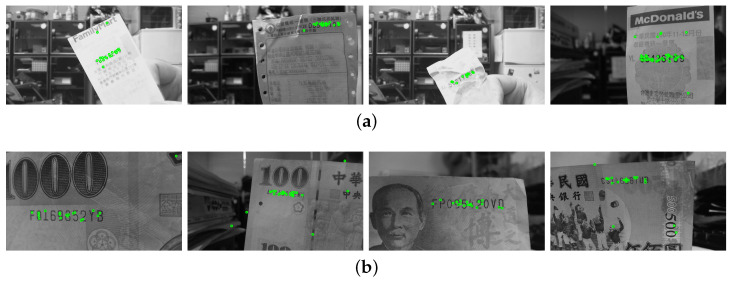
In the first-stage results, the SIFT features are extracted based on the correspondence matching with the reference dataset images. The green circles marked in the images indicate the feature point extraction for (**a**) an invoice and (**b**) a banknote.

**Figure 11:**
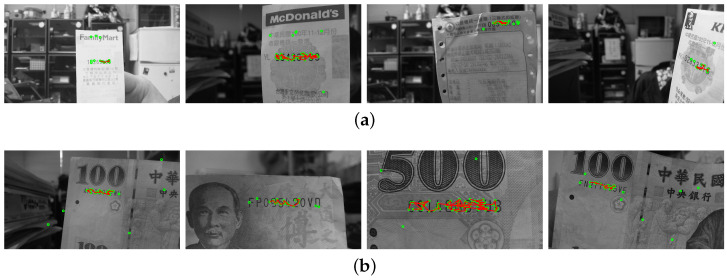
The second-stage results of the OPTICS clustering based on the feature densities, with each individual cluster represented using connected line segments for (**a**) an invoice and (**b**) a banknote.

**Figure 12:**
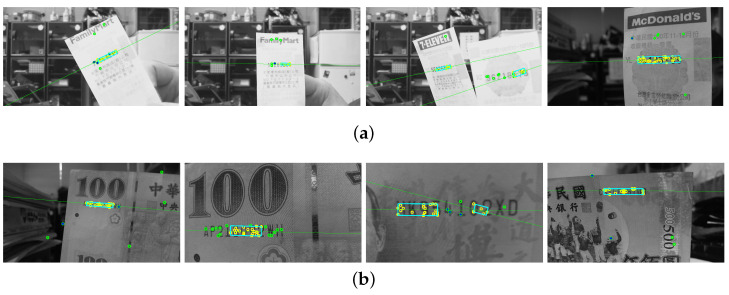
In the third stage, the initial ROI candidate is identified based on the orientation and density of the cluster. The detected bounding box serves as a core region for further expansion in (**a**) an invoice and (**b**) a banknote.

**Figure 13:**
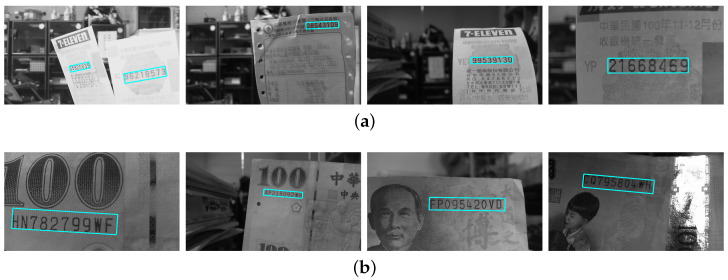
The text region detection results in the last stage, the iterative adjustments are carried out to enlarge the bounding box for the final ROI extraction in (**a**) an invoice and (**b**) a banknote.

The regions of interest in the testing images are recorded with different scales, orientations, illuminations and backgrounds. In the first stage, the SIFT features are extracted based on the correspondence matching with the reference dataset images. The green circles marked in Figure 10a,b illustrate the feature extraction results of the invoice and banknote, respectively. It can be seen that the majority of feature points aggregate around the text regions and with only a small number of outliers. This greatly facilitates feature clustering in the following stage. Figure 11 shows the results of OPTICS clustering based on the feature densities, with each individual cluster represented using connected line segments. From the experiment, the clustering efficiency is demonstrated by the perfect match between the features and text region of interest. In the third stage, the initial ROI candidate is identified based on the orientation and density of the cluster. As illustrated in Figure 12, the detected bounding box serves as a core region for further expansion. The text region detection results in the last stage using iterative adjustments are shown in Figure 13. Since the proposed technique does not take the text boundary into consideration, the enclosing region is set as a rectangular bounding box. Consequently, the image captured with severe perspective distortion might result in a slightly larger region.

Table 2 tabulates the important parameters used in the text region detection. The clustering results for invoice and banknote are tabulated in the third row of Table 1. This indicates that some good results are achieved for invoice and banknote. The evaluation of structured region detection is based on the derivation of regions of interest. Only the detected ROIs that fully cover the text content are considered as a correct result. Since our primary objective is the identification of text regions rather than intermediate feature clustering, we are more interested in the success rates of the ROI detection results. The last two rows of Table 1 tabulate the number of correctly detected regions and the detection accuracy. This shows over 90% of ROI detection rates in the indoor scenes (invoice and banknote). Finally, optical character recognition using the receptors is carried out on the text regions. The performance evaluation for different applications is depicted in Table 3. In general, it takes 2 ms for character recognition using the fast training network.

## 7. Conclusions

In this work, we present a new approach for structured region detection based on correspondence extraction and clustering analysis of local features. The proposed technique is designed for diverse application scenarios. It is capable of dealing with the cases where the target region in different orientations, with size changes, or under perspective distortion. The OPTICS algorithm with clustering density analysis is utilized to derive the characteristics of feature correspondences. Based on the initial ROI candidates identified with cluster orientations, the iterative adjustments are performed to enlarge for text region extraction. The experiments carried out on invoice and banknote have demonstrated the feasibility of the proposed method. Nevertheless, one major limitation of the proposed approach is the detection capability of man-made structures as illustrated in the implementation. In future work, the investigation will be conducted for natural scenes to reveal structural patterns for agriculture applications. The code is available at https://github.com/faketifosi/SCFlow.

## Figures and Tables

**Table 1:** The statistics of experimental results for invoice and banknote applications. The first and second rows indicate the total numbers of testing images and regions of interest, respectively. The numbers of correctly identified clusters and correct regions are shown in the third and fourth rows, respectively.

	Invoice	Banknote
Number of images	113	109
Number of ROIs	116	114
Number of detected clusters	107	106
Number of correct regions	100	102
Accuracy of detection	93%	76%

**Table 2:** The important parameters used for text region detection in our experiments.

Parameter	Description	Value
SIFT distance	The threshold for feature correspondence matching.	0.8
MinPts1	The minimum number of points to form a cluster in OPTICS. (invoice)	#pt/10
MinPts2	The minimum number of points to form a cluster in OPTICS. (banknote and plate)	#pt/6
ϵ	The control parameter for clustering analysis in OPTICS.	0.05
NoRefFeat	The number of reference feature points for corresponding matching.	300
NoHidNode	The number of hidden nodes in the neural network.	100
NoReceptor	The dimension of receptors for the neural network input.	300

**Table 3:** The statistics of character recognition results for the experiments on invoice and banknote.

	Invoice	Banknote
Number of characters	808	949
Correct recognition	673	722
Recognition rate	83%	76%

## Data Availability

Not applicable.
